# A Case Series That Supports the Application of the S2AI Technique for Fractures and Failures After Lumbosacral Fusion

**DOI:** 10.1007/s11420-019-09706-x

**Published:** 2019-07-29

**Authors:** Jeffrey H. Weinreb, Uchechi Iweala, Lauren E. Matteini, Warren D. Yu, Joseph R. O’Brien

**Affiliations:** 1 Department of Orthopaedic Surgery, George Washington University, 2300 M Street, NW, Washington, DC 20037 USA; 2 Department of Orthopaedic Surgery, Loyola University Medical Center, Chicago, IL USA; 3 Washington Spine and Scoliosis Institute, Virginia Hospital Center, Arlington, VA USA

**Keywords:** sacral fractures, lumbosacral fusion, revision surgery, pseudoarthrosis, pelvic instrumentation

## Abstract

**Background:**

Sacral fractures and failures are uncommon after lumbosacral fusion but have received increasing attention in the surgical literature. They can be difficult to diagnose, making timely treatment difficult. No consensus has been reached on the characteristics of these complications or on optimal treatment.

**Questions/Purposes:**

The goal of this retrospective case series is to contribute additional cases of these uncommon complications of lumbosacral fusion to the surgical literature to help clinicians to anticipate, diagnose, characterize, manage, and treat sacral fractures and failures after lumbosacral fusion.

**Methods:**

The medical records of five patients who experienced a sacral fracture or failure after lumbosacral fusion between January 2012 and November 2017 were identified and reviewed retrospectively. Records were reviewed for age, sex, clinical presentation, previous management, outpatient clinical records, imaging, and post-operative course.

**Results:**

Four patients in the series experienced a sacral fracture and one experienced hardware failure. All patients presented with elevated pain and underwent revision surgery. Radiographic detection of the fracture or failure occurred at a mean of 11.2 weeks (range, 3 to 24 weeks) after initial surgery, and the mean age of patients was 68.2 years (range, 63 to 80 years). Of the five patients, four were female; two had been diagnosed with osteoporosis and two with osteopenia. In our case series, the S2–alar–iliac (S2AI) technique was used with success in all five cases.

**Conclusion:**

Fractures and failures after lumbosacral fusion can be difficult to diagnose because of delayed presentation, nonspecific presenting symptoms, and a lack of identifiable mechanism. A high index of suspicion is required to detect these uncommon complications, and patients have responded well to both conservative and surgical treatments.

## Introduction

Sacral fractures and failures are uncommon occurrences after lumbosacral fusion but have received increasing attention in the surgical literature. Several studies have characterized risk factors, outcomes, and radiologic diagnosis, but no consensus has been reached regarding the characteristics of these complications or optimal treatment practices [4, 9, 11, 13, 26, 27]. Sacral fractures and failures after lumbosacral fusion can be difficult to diagnose, making timely management and treatment difficult. Further, sacral fractures can result in a loss of sagittal balance. Research has suggested a number of risk factors that may predispose patients to sacral fracture or failure, including osteoporosis, advanced age, female sex, autologous bone harvesting from the iliac crest, a greater number of segments fused, and sagittal plane imbalance [18, 26] Additionally, obesity has been posited as a risk factor because of the increased load at adjacent segments after multi-segmental fusion [18]. Further, a high pelvic incidence and sacral slope may predispose patients to sacral fracture as a result of high shear stresses across a relatively horizontal sacrum [5, 12]. The purpose of this study is to contribute additional cases of these uncommon complications to the surgical literature to help clinicians to anticipate, diagnose, characterize, manage, and treat sacral fracture and failure after lumbosacral fusion.

## Methods

Records of patients who had experienced a sacral fracture or failure after lumbosacral fusion were retrospectively reviewed. Five patients who experienced a sacral fracture or failure after lumbosacral fusion between January 2012 and November 2017 were included. Records were reviewed for age, sex, clinical presentation, prior management, outpatient clinical records, imaging, and post-operative course. The study was approved by the institutional review board at the primary institution.

## Results

### Case 1

**Fig. 1:**
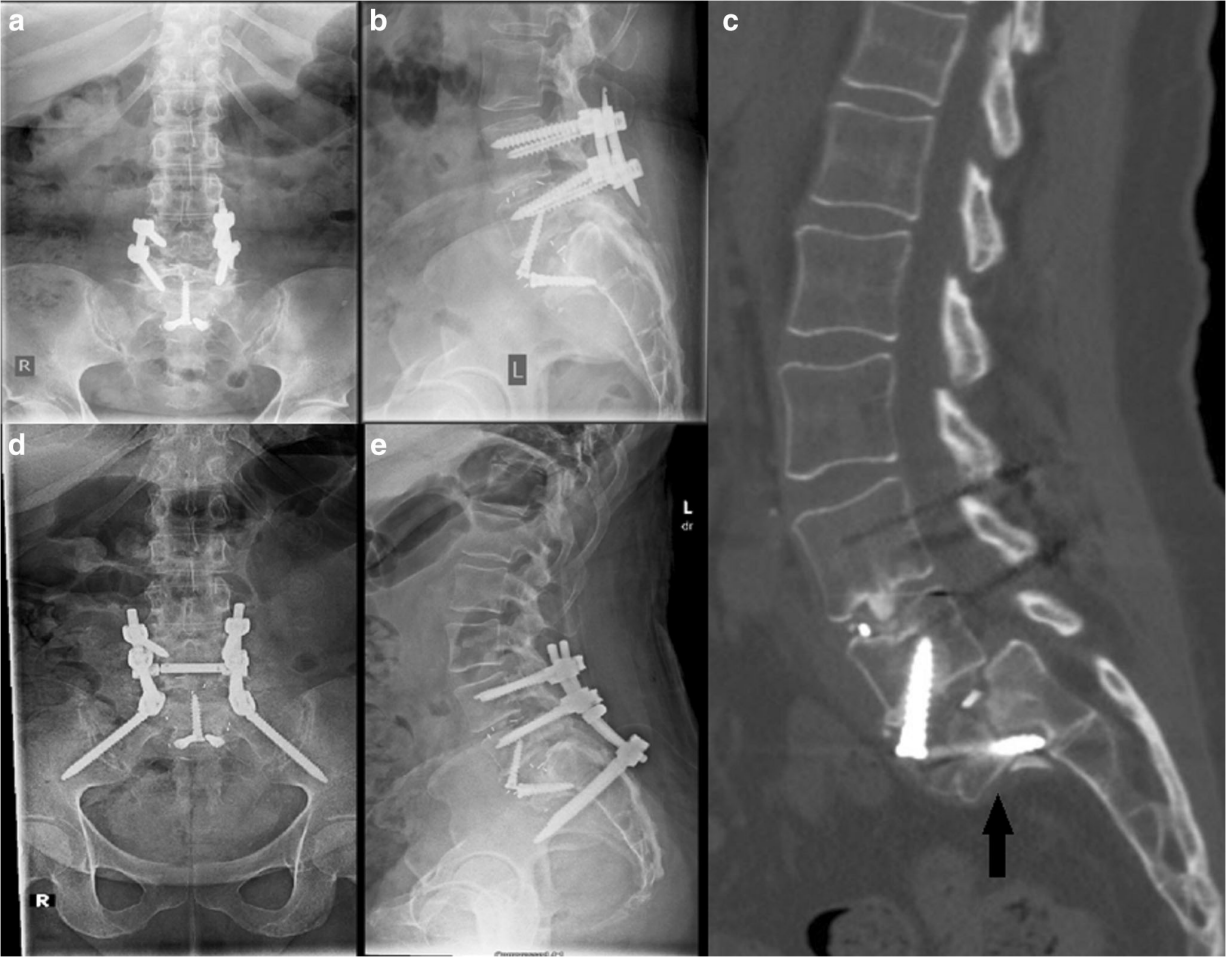
**a** Anteroposterior (AP) and **b** lateral plain radiographs taken before sacral fracture. **c** Representative lateral computed tomographic cut showing sacral fracture (black arrow). **d** AP and **e** lateral post-operative plain radiographs.

A 60-year-old woman with a history of osteopenia presented with low back pain that had been treated with a transforaminal lumbar interbody fusion (TLIF) at L4–L5 6 years before presentation, a microdiskectomy at L5–S1 1 year before presentation, and an anterior lumbar interbody fusion (ALIF) at L5–S1 6 months before presentation. She had pain radiating down both legs in an L5 distribution. Dual-energy X-ray absorptiometry (DEXA) of L1 to L4 and the left femoral neck demonstrated T-scores of − 1.2 and − 1.0, respectively. Plain radiography and computed tomography (CT) performed at the time of presentation revealed a TLIF and bilateral pedicle screw fixation with solid fusion at L4–L5, ALIF cage at L5–S1 with Meyerding grade II spondylolisthesis and pseudoarthrosis, and a sacral end plate fracture (Fig. 1). She underwent revision decompression and fusion, L4 to pelvis; exploration of fusion mass at L4 to S1; and placement of pelvic instrumentation using the S2–alar–iliac (S2AI) technique. The goal of the surgery was to stabilize the fracture and reduce the instability through a solid fusion to limit the irritation of the L5 nerve root. She did well post-operatively, and her pre-operative symptoms were completely relieved at 3 years after her revision surgery.

### Case 2

**Fig. 2:**
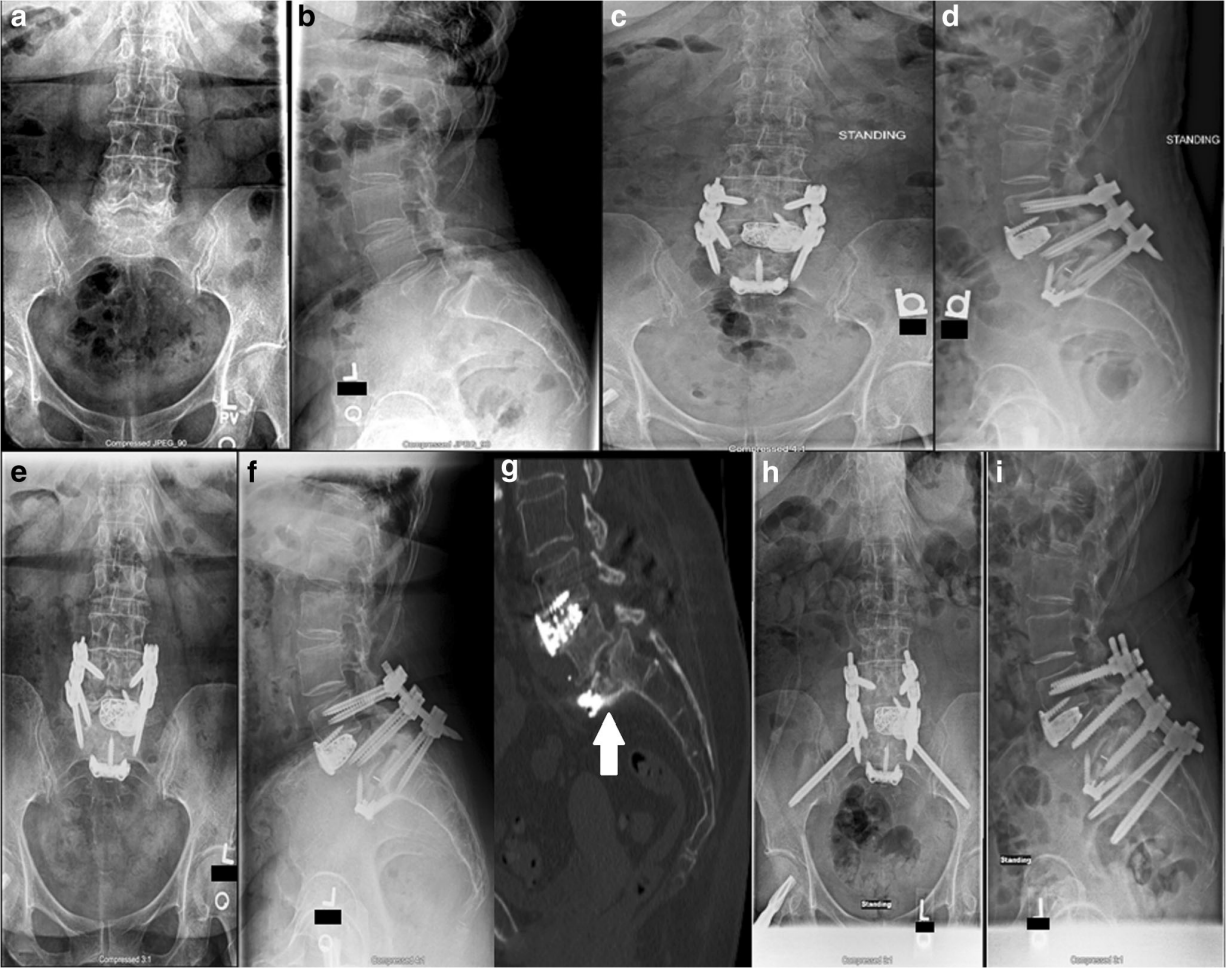
**a** Anteroposterior (AP) and **b** lateral plain radiographs taken before index surgery. **c** AP and **d** lateral plain radiographs taken after index surgery. **e** AP and **f** lateral plain radiographs taken after index surgery. **g** Representative lateral computed tomographic cut showing sacral fracture (white arrow). **h** AP and **i** lateral post-operative plain radiographs.

A 69-year-old woman who presented with low back pain that radiated down the right leg was unable to walk for more than 10 min because of the pain. She also experienced bilateral intermittent toe numbness. A right hip fracture had been treated with cephalomedullary nailing technique, and she was diagnosed with osteoporosis. Her condition had been managed conservatively, with physical therapy, medication, steroid injections, and chiropractic treatment. Steroid injections provided complete but temporary relief. Imaging demonstrated grade I to II spondylolisthesis at L5–S1 with facet arthropathy and neuroforaminal stenosis at L4–L5 and L5–S1. She underwent L4–L5 microdecompression, which alleviated her pain, allowing her to participate in yoga and dancing. However, at approximately 12 months after surgery, her pain began to increase. Imaging done at that time showed no increase in anterolisthesis at L5–S1 but did reveal increased listhesis at L4–L5. Twenty-three months after the index surgery, she was treated with ALIF at L4–L5 and L5–S1, placement of interbody cages at L4–L5 and L5–S1, application of anterior instrumentation at L5–S1, posterior pedicle screw instrumentation from L4 to S1, and posterolateral arthrodesis from L4 to S1. Post-operatively, she experienced complete remission of her neurologic pain until, at 2 months post-operatively, she developed pain with radiation down both legs and difficulty with walking. Plain radiography and CT scanning at that time revealed a fracture through the anterosuperior end plate of S1 (Fig. 2). Twenty-six months after her index surgery and 3 months after her first revision surgery, she underwent exploration of the posterior fusion and removal of instrumentation; revision and decompression of L5–S1; instrumentation placement at the L4–pelvis region; and posterolateral arthrodesis at L4–L5 and L5–S1, with application of pelvic instrumentation using the S2AI technique. Three months post-operatively, she reported 100% improvement in low back pain with some low back soreness, the ability to walk farther, and 80% improvement in neurologic symptoms in the left lower extremity.

### Case 3

**Fig. 3:**
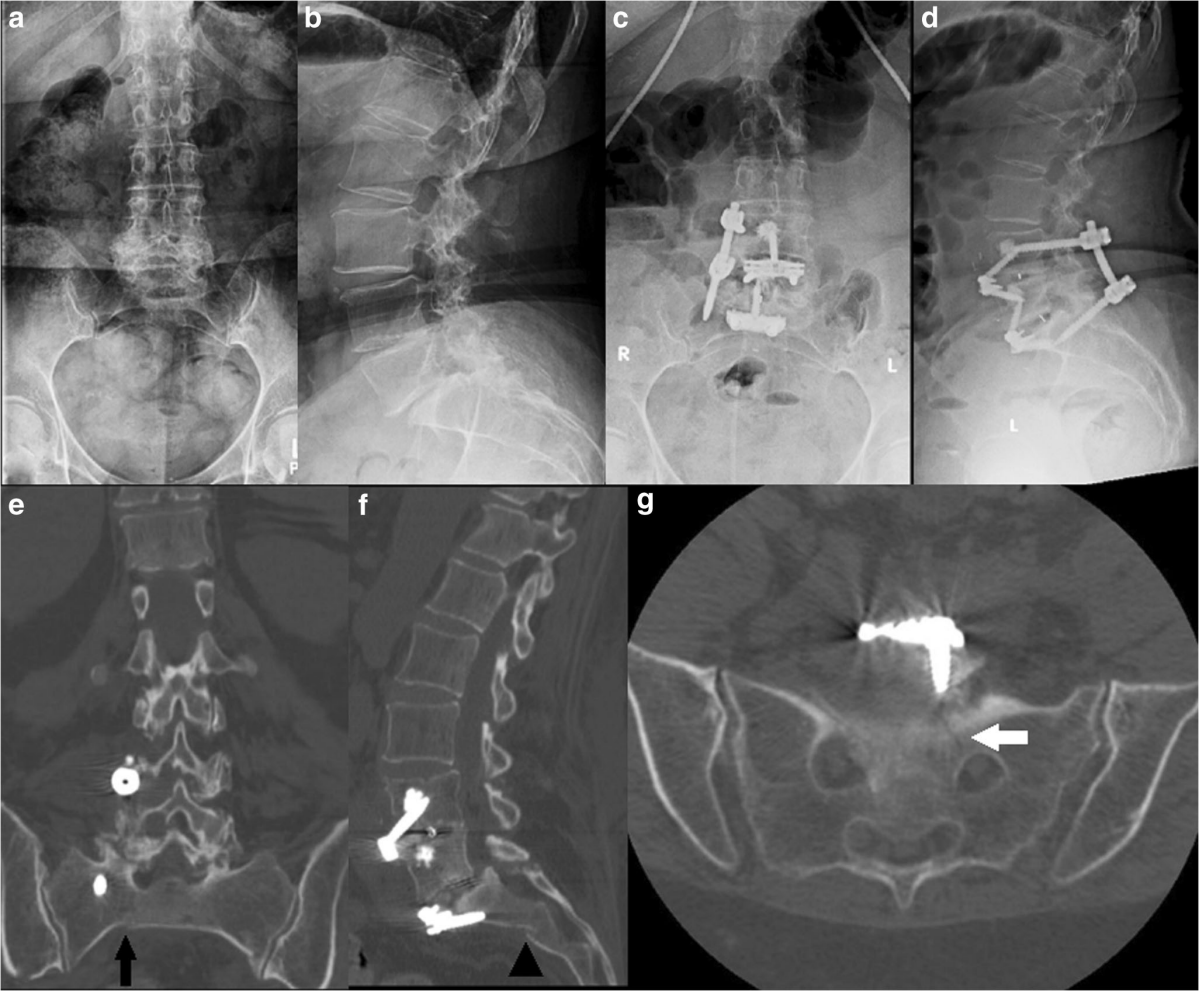
**a** Anteroposterior (AP) and **b** lateral plain radiographs taken before index surgery. **c** AP and **d** lateral plain radiographs taken after index surgery. **e** Representative AP, **f** lateral, and **g** axial computed tomographic cuts showing sacral fracture (black arrow, black arrowhead, white arrow, respectively).

**Fig. 4:**
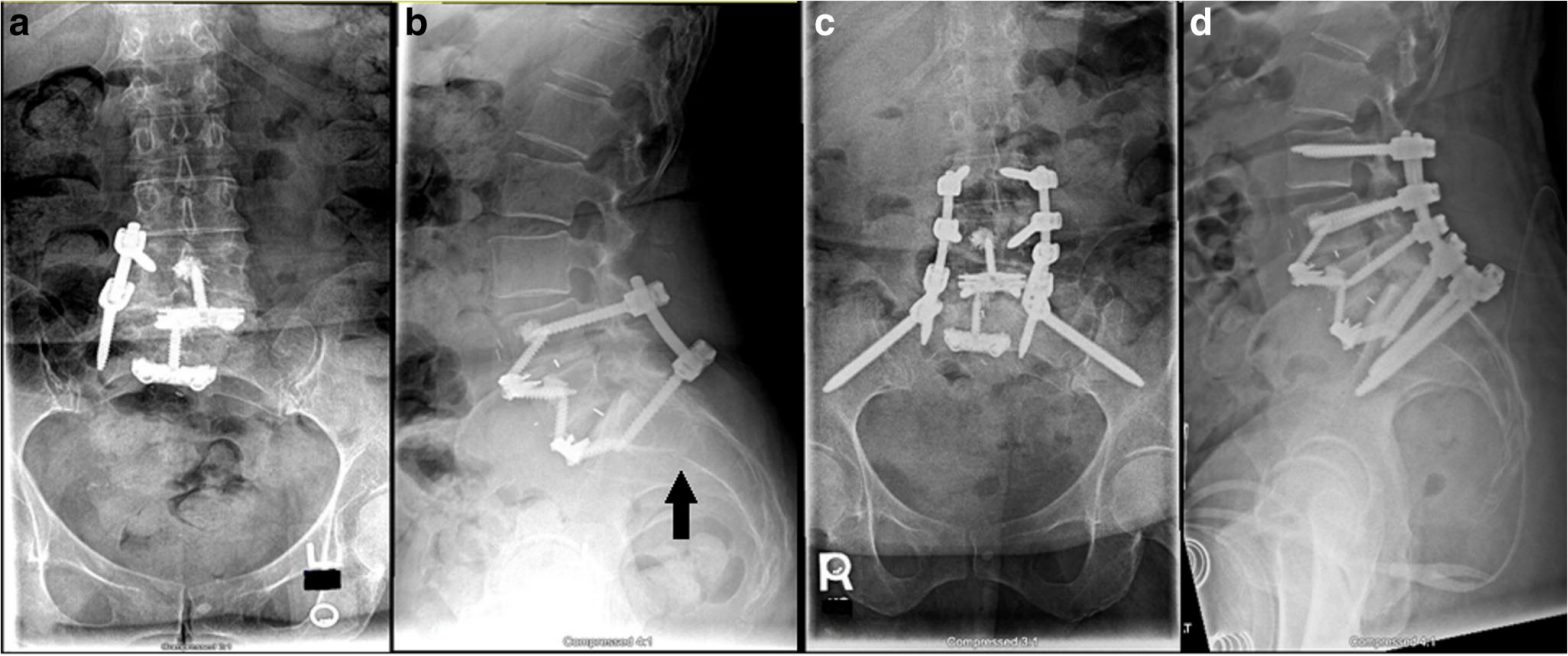
(**a**) Anteroposterior (AP) and (**b**) lateral plain radiographs showing sacral fracture (black arrow). (**c**) AP and (**d**) lateral plain radiographs taken after revision surgery.

A 69-year-old woman with a history of Parkinson’s disorder and osteopenia (DEXA of L1 to L4 and the left femoral neck yielded T-scores of − 2.4 and − 1.0, respectively) presented with pain at the lumbosacral junction that radiated down both legs in an L5 distribution, with more pain in the left leg than in the right. She had been treated with ALIF for realignment and indirect spinal decompression to restore neuroforaminal height. Imaging demonstrated grade I to II spondylolisthesis at L5–S1, severe neuroforaminal stenosis at L4–L5 and L5–S1, and central stenosis at L3–L4. She underwent a right hemilaminectomy at L3–L4 and L4–L5 with undercutting for bilateral decompression, posterior placement of pedicle screws at L4–S1, posterior arthrodesis from L4 to S1, and ALIF with cage placement at L4–L5 and L5–S1. Post-operatively, she reported increasing pain and showed no evidence of having fallen. Plain radiography and CT scanning revealed an H-type sacral fracture to the S1 neural foramen (Figs. 3 and 4). On post-operative day 39, she underwent removal of segmental instrumentation, placement of pelvic instrumentation from L3 to S1 using the S2AI technique, posterolateral arthrodesis from L3 to S1, and decompression of L3–L4 and L4–L5. The patient did well for 2 years but then experienced increasing pain and was found to have lucency around the left iliac screw, despite evidence of good healing otherwise. Fifty-six months after the initial surgery, she underwent removal of instrumentation, with exploration of the posterior fusion, and posterolateral bone grafting of L3–L4, L4–L5, and L5–S1. One year after this revision, the patient reported improvement in pain and was able to participate in physical therapy with oral opioid medication four times per week.

### Case 4

**Fig. 5:**
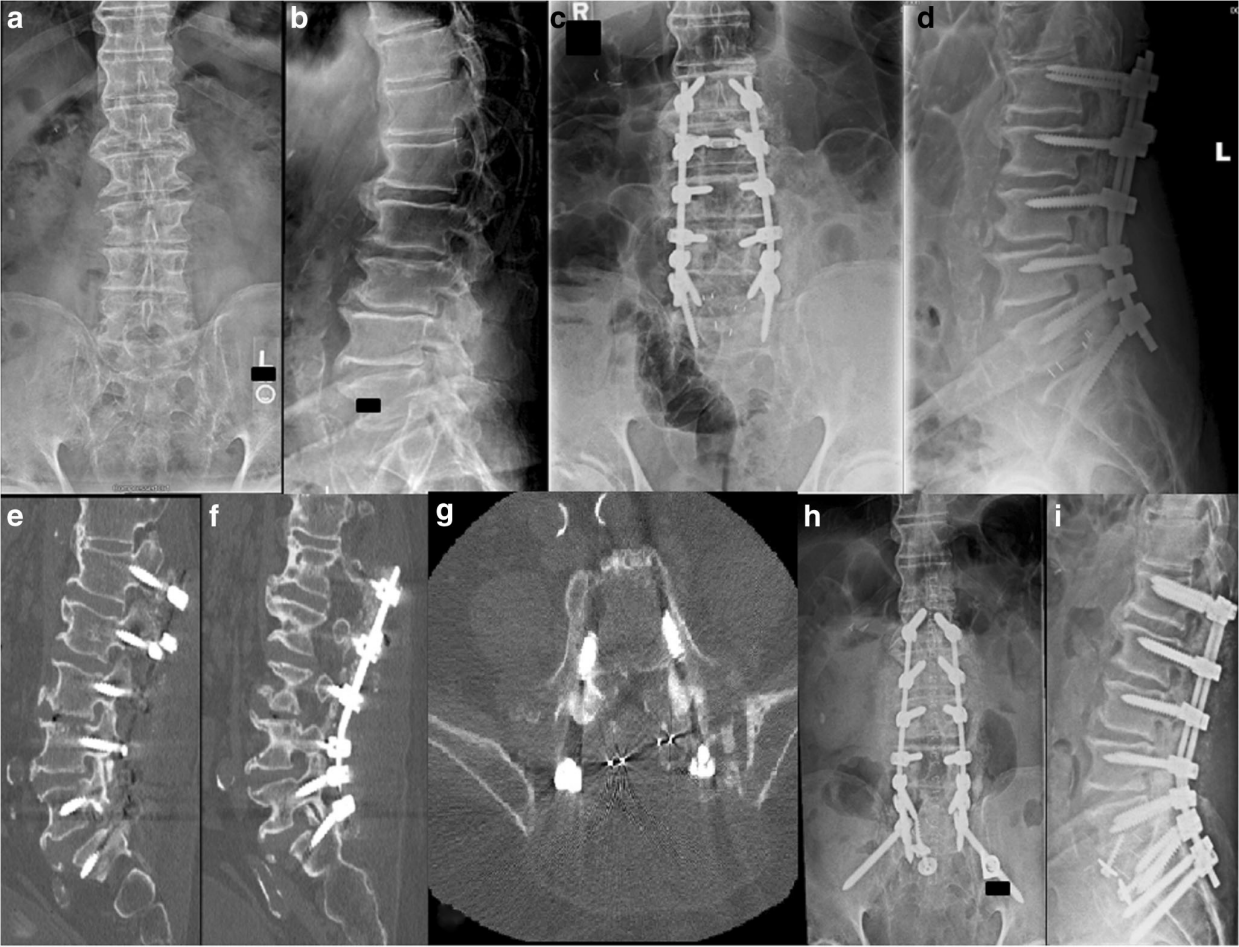
**a** Anteroposterior (AP) and **b** lateral plain radiographs taken before index surgery. **c** AP and **d** lateral plain radiographs taken after index surgery. **e**, **f** Representative lateral computed tomographic (CT) cuts showing hardware pullout. **g** Representative axial CT cut showing hardware pullout and cage extrusion into canal. **h** AP and **i** lateral post-operative plain radiographs.

An 80-year-old man presented with symptoms that included low back pain that radiated down both anterior thighs and posterior legs. He had a protracted history of low back pain previously managed conservatively, followed by a lumbar microdiskectomy. Plain radiographs revealed extensive degenerative disk disease with neuroforaminal stenosis of the lower lumbar spine, most pronounced at L3–4, L4–5, and L5–S1, and grade I retrolisthesis of L3 on L4. Smith–Peterson osteotomies of L2–L3, L3–L4, L4–L5, and L5–S1 with posterior interbody fusion of L5–S1, posterior segmental instrumentation of L5–S1, posterior arthrodesis of L1–S1, and revision decompression of L1–S1 were performed. The patient initially reported improvement in pain post-operatively but noted gradually increasing pain with ambulation. Three weeks after surgery, CT scanning revealed vertebral fracture, instrumentation pullout, and posterior interbody cage extrusion into the spinal canal (Fig. 5). The patient then underwent a staged procedure with removal of hardware related to L1-to-S1 and L5–S1 posterior interbody fusion with allograft and demineralized bone matrix revision; posterolateral fusion from T12 to pelvis; instrumentation placement from T12 to pelvis using the S2AI technique; and increases in the diameter of all screws on post-operative day 20, followed by ALIF of L5–S1 with interbody cage and anterior instrumentation on post-operative day 25. The patient reported reduced pain at 1 year after surgery but was progressively unable to walk because of metastatic prostate cancer.

### Case 5

**Fig. 6:**
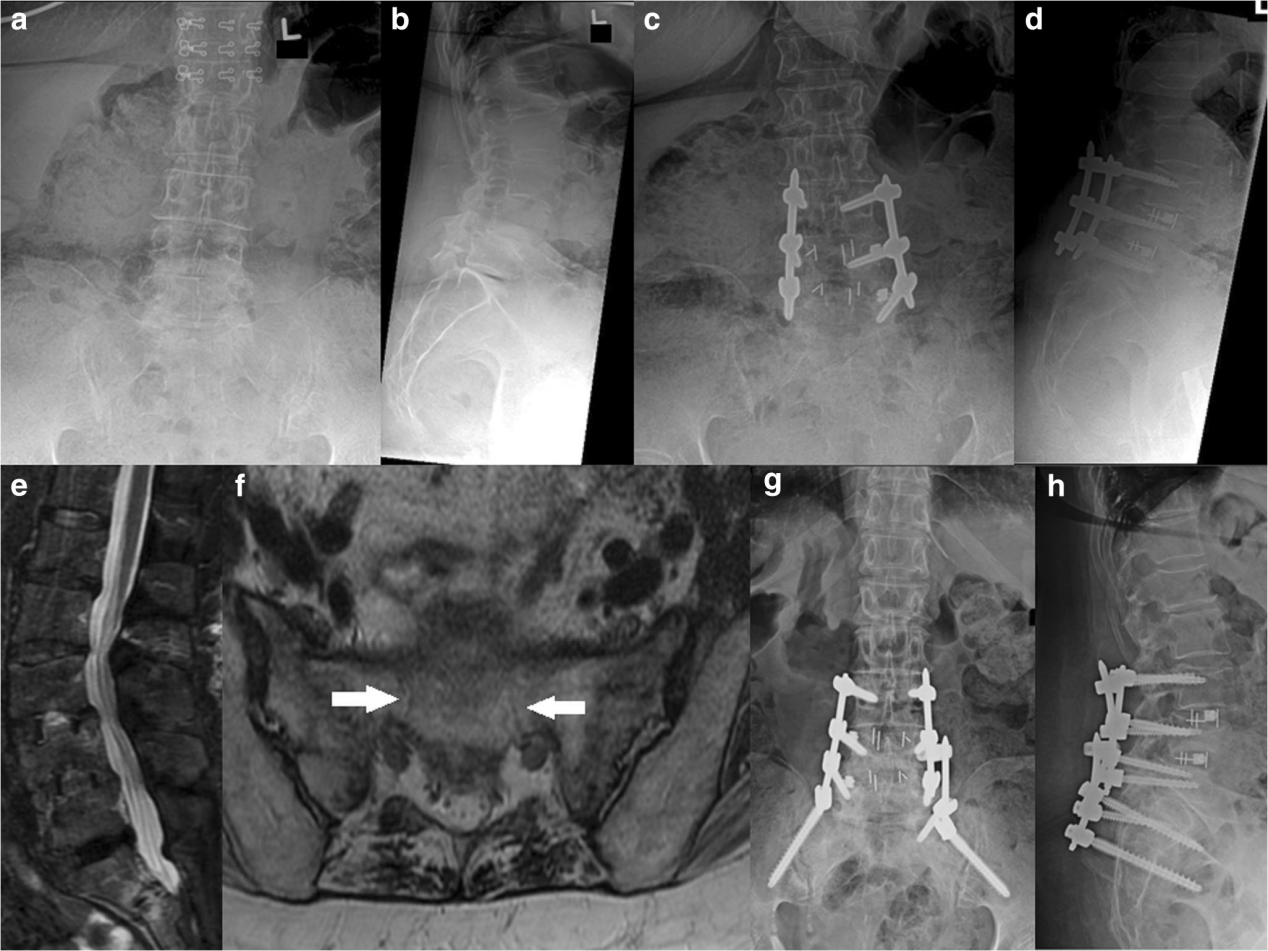
**a** Anteroposterior (AP) and **b** lateral plain radiographs taken before index surgery. **c** AP and **d** lateral plain radiographs taken after index surgery. **e** Representative AP and **f** coronal magnetic resonance imaging cuts showing sacral fractures (white arrows). **g** AP and **h** lateral plain radiographs taken after revision surgery.

A 63-year-old woman with a history of left hip fracture that had been repaired with open reduction and internal fixation of the femoral neck presented with increasing left leg sacral neuritis in an L5 distribution. DEXA of L1 to L4 and the left femoral neck yielded T-scores of − 1.6, although the patient’s history of hip fracture suggested a diagnosis of osteoporosis. Her initial presenting symptoms included lower back pain radiating down the left leg that progressed over 4 years. Her care had been managed conservatively, with physical therapy, medication, and multiple epidural steroid injections that provided temporary relief. Radiographic imaging showed anterolisthesis of L3–L4 and L4–L5. She underwent ALIF from L3 to L5 with improvement in pre-operative symptoms but experienced increasing pain post-operatively. Magnetic resonance imaging and plain radiography performed 17 weeks after the index operation revealed an H-type fracture of S1 and S2 (Fig. 6). Five months after the index operation, she underwent exploration of the posterior fusion and removal of instrumentation, with re-instrumentation of L3–pelvis region with iliac screws. At her 3-week post-operative visit, she reported reduced pain, minimal analgesic use, and good ambulation.

## Discussion

Sacral fractures and failures after lumbosacral fusion have been described in the literature as being difficult to detect, with delayed diagnosis in almost every patient [26]. The difficulty in detecting sacral fractures and failures after lumbosacral fusion is likely a result of post-operative pain being so common that it is not necessarily seen as an indication for advanced imaging. Post-operative imaging protocols differ according to a surgeon’s preference, and not all patients present with a specific inciting factor such as a fall or other trauma. In one study of 16 patients (mean age, 66.4 years; range, 36 to 79 years), sacral fractures were not detected for an average of 5 weeks (range, 1 to 49 weeks) [26]. This is similar to our series, in which detection did not occur until an average of 11.2 weeks (range, 3 to 24 weeks [mean age of patients, 68.2 years; range, 63 to 80 years]). Osteoporosis has been suggested as a risk factor for sacral fracture after lumbosacral fusion [26]. Advanced age and female sex have also been suggested as risk factors, but fractures in these cases are likely related to reduced bone mineral density [26]. A horizontal fracture through the sacral body, involving the screw holes, has been suggested as the prototypical fracture pattern seen in sacral fractures after lumbosacral fusion [27]. In the current study, two patients had a history of osteoporosis, two had a history of osteopenia, four were female, and three initially presented with spondylolisthesis (Table 1).

**Table 1:** Patient demographics and clinical characteristics

Case no.	Sex	Age	Segments	Bone graft	Weeks until diagnosis	Indication	New construct
1	Female	60	2	BMP, local autograft	24	S1 end plate fracture	L4 to pelvis
2	Female	69	2	BMP, local autograft	8	S1 end plate fracture	L4 to pelvis
3	Female	69	2	BMP, local autograft	4	S1 H-type fracture	L4 to pelvis
4	Male	80	5	DBM	3	Cage extrusion into canal, instrumentation pullout	T12 to pelvis
5	Female	63	2	DBM	17	S1–S2 H-type fracture	L3 to pelvis

*BMP* bone morphogenetic protein, *DBM* demineralized bone matrix

There is no consensus on optimal treatment for sacral fracture or failure after lumbosacral fusion. Three out of four cases reported by Vavken et al. were treated conservatively with good results, whereas all five cases described by Papadopoulos et al. were treated with revision surgery, also with good results [18, 26]. In a radiographic study conducted by Wilde et al., 11 of 23 fractures were treated nonoperatively, and the other 12 went on to surgery with transiliac fixation [27]. In our study, all five cases were treated with revision surgery. All patients initially did well after surgery, but in case 3, the patient was found to have asymptomatic lucency around the left iliac screw and underwent an additional revision 2 years after her first, and in case 4, the patient experienced worsening ambulation as a result of metastatic prostate cancer.

In patients undergoing fusion from the thoracic spine to L5, distal fixation has been advocated as a way to prevent subsequent L5–S1 degenerative disk disease and loss of sagittal alignment [1, 2]. However, when compared with fusions to L5, long fusion to the sacrum has been associated with a higher rate of complications, including pseudarthrosis, sacral fracture, and peri-operative medical morbidity [1, 2, 10].

When extending a construct distal to S1, several options exist, including S2 screws, iliac fixation, and S2AI fixation. In contrast to S2AI fixation, S2 screws have been advocated as an option to protect S1 pedicle screws but have also been shown to have the poorest pullout strength in the sacrum because they have no iliac purchase [12, 29]. Additionally, S2–alar screws are dorsal to the lumbosacral pivot point, which is defined as the point at the middle osteoligamentous column between the last lumbar vertebra and the sacrum. Ideally, an implant will extend anterior to the pivot point because studies have shown that this extends the time to construct failure in flexion and construct stiffness, as is the case with iliac fixation [14].

**Fig. 7:**
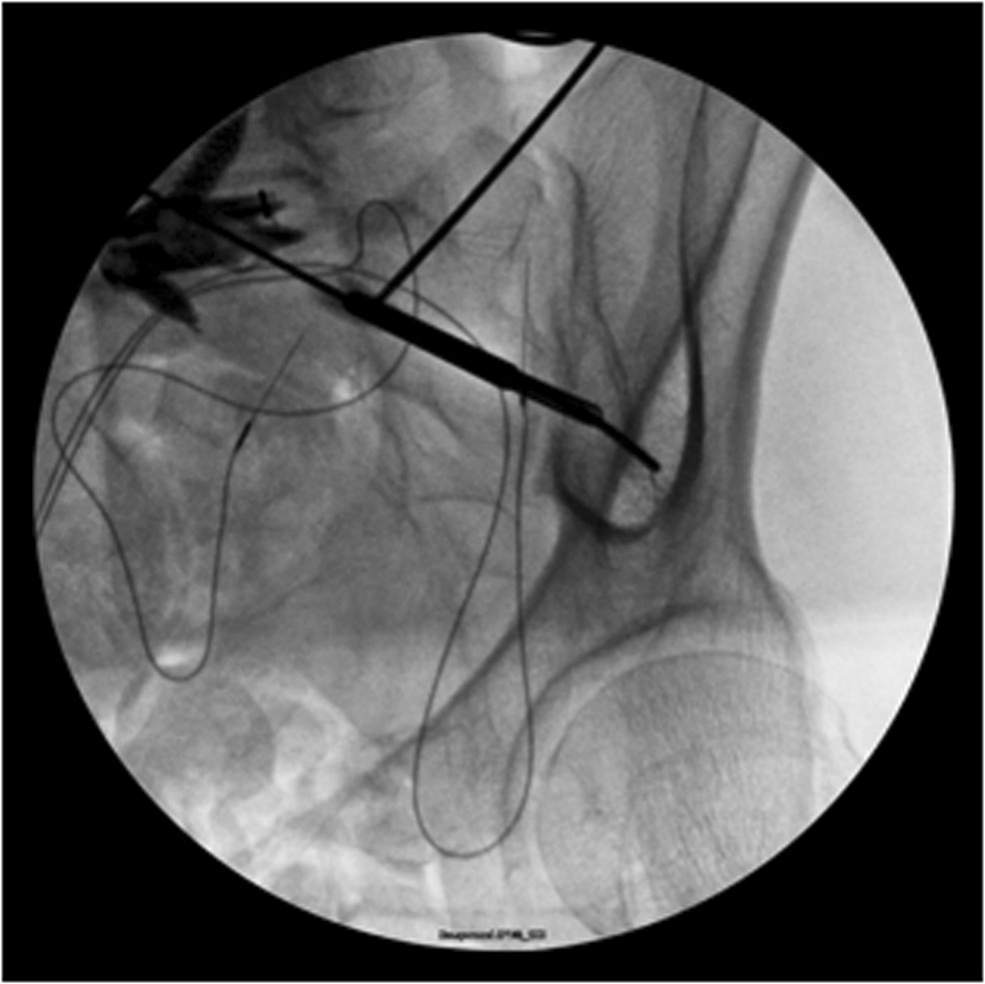
The radiographic teardrop that represents the radiographic confluence of the posterior superior iliac spine, the sciatic notch, and the anterior inferior iliac spine.

To achieve optimal iliac fixation, screws with enough diameter and length to achieve the greatest pullout strength should be used, along with intra-operative fluoroscopic landmarks for secure placement. Generally, screw trajectory should be based on the teardrop X-ray view, which is the radiographic confluence of the posterior superior iliac spine (PSIS), the sciatic notch, and the anterior inferior iliac spine (Fig. 7) [21]. Optimal lumbopelvic fixation can be achieved with screws that are 6 to 8 mm in width with a length of up to 130 and 140 mm in female and male patients, respectively [21].

Iliac screws have been shown to protect S1 screws without sacroiliac degeneration on 5 to 10 years of follow-up [25]. Additionally, iliac screws may reduce the volume of autologous bone graft required but may need to be removed because of pain or prominence [19, 20, 24]. Tsuchiya et al. found that 23 of 67 patients with iliac screws underwent removal within 5 to 10 years because of either prominence or the surgeon’s choice [25].

S2AI fixation can be performed using either an open or minimally invasive approach [8, 16]. To insert an S2AI screw, the surgeon selects a starting point 1 mm inferior and 1 mm lateral to the S1 dorsal foramen. The surgeon then advances a 2.5-mm drill through the sacral ala with the drill directed inferiorly toward the greater trochanter and laterally with approximately 40 to 50° of angulation relative to the horizontal line connecting the PSIS and 20 to 30° caudal from straight lateral [15]. S2AI screws are biomechanically as stable as test constructs using iliac screws; O’Brien et al. found that S2AI screws of 65 to 80 mm in length were biomechanically equivalent to 90-mm iliac screws [17]. Using the S2AI technique, 40 to 50 mm of the screw has sacral purchase, and the remainder of screw is within the ilium [15, 28]. Because S2AI screws are 15 mm deeper than iliac screws, S2AI screw fixation results in less-prominent hardware than does iliac screw fixation [23]. Sponseller et al. found no instances of deep infection, implant prominence, late skin breakdown, or anchor migration in 26 patients undergoing S2AI screw fixation, as compared with three instances of deep wound infection; two instances of superficial wound infection; and one instance each of implant prominence, late skin breakdown, and anchor migration in 26 cases of traditional iliac screw fixation [23]. It is worth noting that in one cadaveric study, 60% of S2AI screws were shown to violate the articular cartilage of the sacroiliac joint [15]. However, outcomes in 52 adult and pediatric patients showed no increase in sacroiliac joint pain at follow-up (a mean of 2.5 years) [8].

Smith et al. found that 86 patients without pre-operative pseudoarthrosis who underwent S2AI screw fixation had a 95.3% fusion rate at 2 to 5 years of follow-up [22]. Interestingly, 10.4% of patients had evidence of S2 loosening on imaging, including two cases that required hardware removal and one that required S2AI instrumentation exchange after pseudarthrosis developed. The authors have had good results using the S2AI technique to obtain sacropelvic fixation for both simple fusion and instrumentation placement, as well as for fractures. Current literature indicates that the S2AI technique is superior to other methods for spinopelvic fixation [3, 6, 7]. Further studies may focus on comparisons of the S2AI technique with traditional modes of sacropelvic fixation in the context of traumatic or post-operative fracture.

In conclusion, sacral fractures and failures after lumbosacral fusion are an uncommon occurrence after lumbosacral fusion but have been reported with increasing frequency in the spine surgery literature. Although prototypical fracture patterns, patient demographics, and risk factors have been described, no two cases are identical. According to research suggesting that an increased sacral slope or pelvic incidence may increase the risk of sacral fracture after instrumentation placement and fusion, sagittal balance is an important pre- and post-operative consideration. Surgeons may consider obtaining standing anteroposterior and lateral projections to measure the pelvic parameters. Patients have responded well to both conservative management and surgical revision, and in our case series the S2AI technique was used with success in all five cases. Finally, clinicians should maintain a high index of suspicion for sacral fracture or failure because it often has nonspecific presenting symptoms, no identifiable underlying mechanism, and delayed presentation.
